# Neurophysiological and Psychophysical Biomarkers for Predicting Spinal Cord Stimulation Treatment Response in Chronic Neuropathic Pain: A Systematic Review

**DOI:** 10.3390/jpm16100486

**Published:** 2026-09-22

**Authors:** Charles A. Odonkor, Richard Fagbemigun, Fatimah B. Alhassan, Alaa Abd-Elsayed

**Affiliations:** 1 Department of Orthopaedics and Rehabilitation, Yale School of Medicine, Division of Physiatry, Interventional Pain Medicine, Yale University, 47 College Street, New Haven, CT 06511, USA; 2 Department of Orthopaedics and Rehabilitation, Division of Physiatry, Yale New Haven Hospital, 20 York Street, New Haven, CT 06510, USA; 3 Department of Physical Medicine and Rehabilitation, Johns Hopkins University School of Medicine, Baltimore, MD 21287, USA; rfagbem1@jh.edu; 4 Department of Internal Medicine, Lehigh Valley Health Network, Allentown, PA 18102, USA; fxa048@jefferson.edu; 5 Department of Anesthesiology, School of Medicine and Public Health, University of Wisconsin, Madison, WI 53705, USA; abdelsayed@wisc.edu

**Keywords:** spinal cord stimulation, neuropathic pain, biomarkers, treatment response prediction, quantitative sensory testing, conditioned pain modulation, electroencephalography, evoked potentials, personalized neuromodulation

## Abstract

**Background:** Spinal cord stimulation (SCS) is an established therapy for chronic neuropathic pain, yet a substantial minority of implanted patients do not obtain durable relief, and patient selection still relies largely on a subjective percutaneous stimulation trial. Objective neurophysiological and psychophysical biomarkers could enable precision selection, but their predictive value has not been consolidated across modalities. **Objectives:** To evaluate whether pre-treatment or intraoperative neurophysiological (electroencephalography (EEG), magnetoencephalography (MEG), somatosensory evoked potentials (SSEP), evoked compound action potentials (ECAP)) and psychophysical (conditioned pain modulation (CPM), quantitative sensory testing [QST]) biomarkers predict SCS treatment response in adults with chronic neuropathic pain. **Methods:** Following a pre-registered protocol (International Prospective Register of Systematic Reviews (PROSPERO CRD420261410302)) and the Preferred Reporting Items for Systematic Reviews and Meta-Analyses (PRISMA) 2020 guideline, we searched six databases and two registries; the final search was run on 4 June 2026. Records underwent dual, independent screening in Rayyan; data were extracted in duplicate and risk of bias was assessed with the Quality In Prognosis Studies (QUIPS) tool, the Prediction model Risk Of Bias Assessment Tool (PROBAST), or the Quality Assessment of Prognostic Accuracy Studies (QUAPAS) tool. Because of clinical and methodological heterogeneity, findings were synthesized narratively using the Synthesis Without Meta-analysis (SWiM) guideline. **Results:** Twelve peer-reviewed studies met eligibility. Most were prognostic-factor or responder-discrimination studies rather than prospectively validated predictors; two were multivariable machine learning prediction models and one used ECAP-derived neural dose as a therapy-embedded surrogate. The strongest single-study signal was the preoperative SSEP (one 2003 cohort, *n* = 95), in which normal dorsal-column central conduction was associated with a 75% success rate; this legacy finding requires replication with contemporary SCS paradigms. Resting EEG/MEG spectral features (alpha, gamma) discriminated responders in several small cohorts. The two prediction models reported apparent internal accuracy of 76–88% (area under the curve up to 0.88) but neither underwent external validation. QST and CPM findings were promising but directionally inconsistent. All 12 studies were at high overall risk of bias. **Conclusions:** Objective biomarkers—most notably the preoperative SSEP and resting-state EEG/MEG spectral features—show promise for predicting SCS response, but the evidence is preliminary, heterogeneous, small, and lacks external validation. Adequately powered, prospective, externally validated studies are needed before clinical adoption.

## 1. Introduction

Chronic neuropathic pain affects a large and growing population and is frequently refractory to pharmacological and conservative management [1,2]. Spinal cord stimulation (SCS) is a well-established neuromodulation therapy for several neuropathic conditions, including failed back surgery syndrome (FBSS)/persistent spinal pain syndrome, complex regional pain syndrome (CRPS), painful diabetic polyneuropathy, and radicular pain [3,4,5]. Despite technological advances including high-frequency, burst, and closed-loop paradigms, a proportion of implanted patients do not achieve durable, clinically meaningful relief, exposing non-responders to the cost, risk, and reduced quality of life associated with an ineffective implanted device [4,5].

Current patient selection depends heavily on a percutaneous stimulation trial and on subjective or psychological measures [6,7,8]. The trial is a functional read-out of the therapy rather than a mechanism-based biomarker, and it carries false-positive and false-negative rates as well as procedural risk; to date, it has not been replaced by any validated objective test [8]. This gap has driven interest in objective, mechanism-informed biomarkers—measured before implantation or intraoperatively—that could identify likely responders in advance [9,10,11]. Two broad classes are especially promising: neurophysiological measures (electroencephalography [EEG], magnetoencephalography [MEG], somatosensory evoked potentials [SSEP], and evoked compound action potentials [ECAP]) and psychophysical measures of pain processing (quantitative sensory testing [QST] and conditioned pain modulation [CPM]) [9,10,11].

Chronic pain is biologically and clinically heterogeneous, and this heterogeneity is a central obstacle to effective treatment: an intervention that benefits one patient may fail in another. Personalized—or precision—pain medicine seeks to overcome this by matching therapy to an individual’s underlying pain biology, drawing on molecular, neurophysiological, and imaging biomarkers, omics technologies, and artificial intelligence to move beyond a one-size-fits-all approach. Objective neurophysiological and psychophysical biomarkers are attractive within this framework because they can be acquired before or during the SCS trial and are grounded in the pain-processing and central-sensitization mechanisms that SCS is thought to engage.

Although individual studies have reported associations between such biomarkers and SCS outcomes, the evidence has not been systematically consolidated across modalities, outcomes, and study designs [12,13,14]. We therefore conducted a systematic review to evaluate whether pre-treatment or intraoperative neurophysiological and psychophysical biomarkers predict SCS treatment response in adults with chronic neuropathic pain, to characterize the direction and consistency of associations by biomarker domain, and to appraise the methodological quality and translational readiness of this literature.

## 2. Materials and Methods

### 2.1. Protocol and Registration

This review was prospectively registered (International Prospective Register of Systematic Reviews (PROSPERO) CRD420261410302) and is reported in accordance with the Preferred Reporting Items for Systematic Reviews and Meta-Analyses (PRISMA) 2020 statement [15]. Deviations from the registered protocol are documented in Section 2.8.

### 2.2. Review Question, PICO Framework, and Eligibility Criteria

The review question was: in adults with chronic neuropathic pain undergoing or being evaluated for SCS, do objective pre-treatment or intraoperative neurophysiological or psychophysical biomarkers predict treatment response? Because the exposure is a prognostic factor rather than an intervention, we used a Population, Intervention, Comparator, Outcome (PICO) framework adapted for prognosis:

- Population (P): adults (≥18 years) with chronic neuropathic pain (≥3 months), defined clinically by validated screening tools (PainDETECT, DN4), or by International Association for the Study of Pain (IASP) grading—including FBSS/persistent spinal pain syndrome, CRPS, painful diabetic polyneuropathy, post-herpetic neuralgia, and other peripheral neuropathic pain—undergoing or being evaluated for SCS (trial or permanent).
- Index factor/exposure (I): an objective neurophysiological (EEG, MEG, SSEP/evoked potentials, ECAP) or psychophysical (QST, CPM) biomarker measured before treatment or intraoperatively.
- Comparator (C): SCS responders versus non-responders (as defined by each study), biomarker strata (e.g., normal versus abnormal), or association with a continuous outcome.
- Outcome (O): SCS treatment response—most commonly ≥50% (or ≥30%) pain reduction, successful trial-to-implant conversion, or study-defined responder status.
- Timing/setting: biomarker acquired pre-treatment or intraoperatively; clinical settings delivering or evaluating SCS.

We excluded studies in which only the effect of SCS on a biomarker was reported (mechanistic or monitoring studies, addressed in a companion scoping review); studies whose predictors were exclusively clinical, demographic, or psychological; non-SCS neuromodulation; animal or in silico studies; case series with fewer than five participants; registry protocols without results; and non-peer-reviewed conference abstracts. No language or date restrictions were applied. Biomarkers acquired only during or after therapy (for example, closed-loop ECAP feedback) were not eligible as pre-treatment predictors and, where retained, are reported separately as therapy-embedded surrogate-response measures.

### 2.3. Information Sources and Search Strategy

We searched PubMed/MEDLINE, Embase (Ovid), Scopus, Web of Science Core Collection, Cochrane CENTRAL, and APA PsycINFO, together with ClinicalTrials.gov and the WHO International Clinical Trials Registry Platform. Databases were searched from inception, and the final search was executed on 4 June 2026. The strategy combined three concept blocks—spinal cord stimulation, the target biomarkers, and prediction/response—using controlled vocabulary and free-text terms adapted to each interface; the complete, database-specific strategies and per-source record yields are provided in Supplementary File S1. The search strategy was developed with a medical research librarian and peer-reviewed using the Peer Review of Electronic Search Strategies (PRESS) 2015 guideline [16]. Reference lists of included studies and relevant reviews were hand-searched, and forward citation searching was performed.

### 2.4. Study Selection

Records were de-duplicated and screened in Rayyan (Rayyan Systems, Inc., Cambridge, MA, USA) [17]. Two reviewers (R.F. and F.A.) independently screened titles/abstracts and then full texts, blinded to each other’s decisions, with a third reviewer (C.A.O.) arbitrating disagreements. Chance-corrected agreement (Cohen’s kappa) was quantified at two stages. First, during a pre-screening calibration exercise on a shared random sample of 100 records, the reviewers reached kappa = 0.94 (almost perfect), confirming a shared understanding of the criteria before full screening began. Second, across the subsequent title/abstract screening sample of 206 records, agreement was kappa = 0.61 (substantial; 92.2% raw agreement); the lower value reflects the more heterogeneous, borderline records encountered once screening extended beyond the calibration set, and all disagreements were resolved by consensus.

### 2.5. Data Extraction and Handling of Multiple Reports

Two reviewers (R.F. and F.A.) independently extracted data using a piloted data-extraction form informed by the CHecklist for critical Appraisal and data extraction for systematic Reviews of prediction Modeling Studies (CHARMS) and the Transparent Reporting of a multivariable prediction model for Individual Prognosis Or Diagnosis (TRIPOD) statement [18] capturing study design, setting, population and pain etiology, SCS paradigm, biomarker modality and timing, biomarker parameters and thresholds, responder definitions, outcome timing, and univariable and multivariable associations; for prediction-model studies, we additionally recorded model type, predictor selection, sample adequacy, internal and external validation, and calibration. Where two or more reports (including conference abstracts and their subsequent full publications) described the same cohort, they were linked and counted once, and the most complete peer-reviewed report was used for extraction, so that no participants were double-counted.

### 2.6. Risk of Bias Assessment

Risk of bias was assessed in duplicate and tailored to study type: the Quality In Prognosis Studies (QUIPS) tool for prognostic-factor and responder-discrimination studies [19], the Prediction model Risk Of Bias Assessment Tool (PROBAST) for multivariable prediction-model studies [20], and the Quality Assessment of Prognostic Accuracy Studies (QUAPAS) tool where a study was framed as prognostic (test) accuracy [21]. To permit a single visual summary (Figure 1), tool-specific domains were harmonized to six positions: D1 (participants/participation), D2 (attrition/predictors), D3 (biomarker/predictor measurement), D4 (outcome), D5 (confounding), and D6 (analysis and reporting). The four PROBAST/QUAPAS domains map to D1–D4, with D5–D6 marked as not applicable. Each domain was rated low, moderate, high, or unclear; where reviewers differed, the higher-risk rating was retained; and the overall judgment for a study was the highest risk level assigned to any of its applicable domains (i.e., any high-risk domain yields an overall high-risk rating), consistent with QUIPS/PROBAST guidance.

### 2.7. Synthesis

We pre-specified a random-effects meta-analysis where at least three studies reported commensurate biomarkers, thresholds, and outcome definitions. In this case, no biomarker domain met this condition: studies differed in modality, specific parameter, responder threshold (≥30% vs. ≥50% reduction vs. trial-to-implant conversion), and timing, and most reported univariable associations rather than adjusted effects. Meta-analysis was therefore judged inappropriate, and we synthesized findings narratively following the Synthesis Without Meta-analysis (SWiM) guideline [22], grouping studies by biomarker domain and summarizing the direction, magnitude, and consistency of biomarker-response associations. 

**Figure 1:**
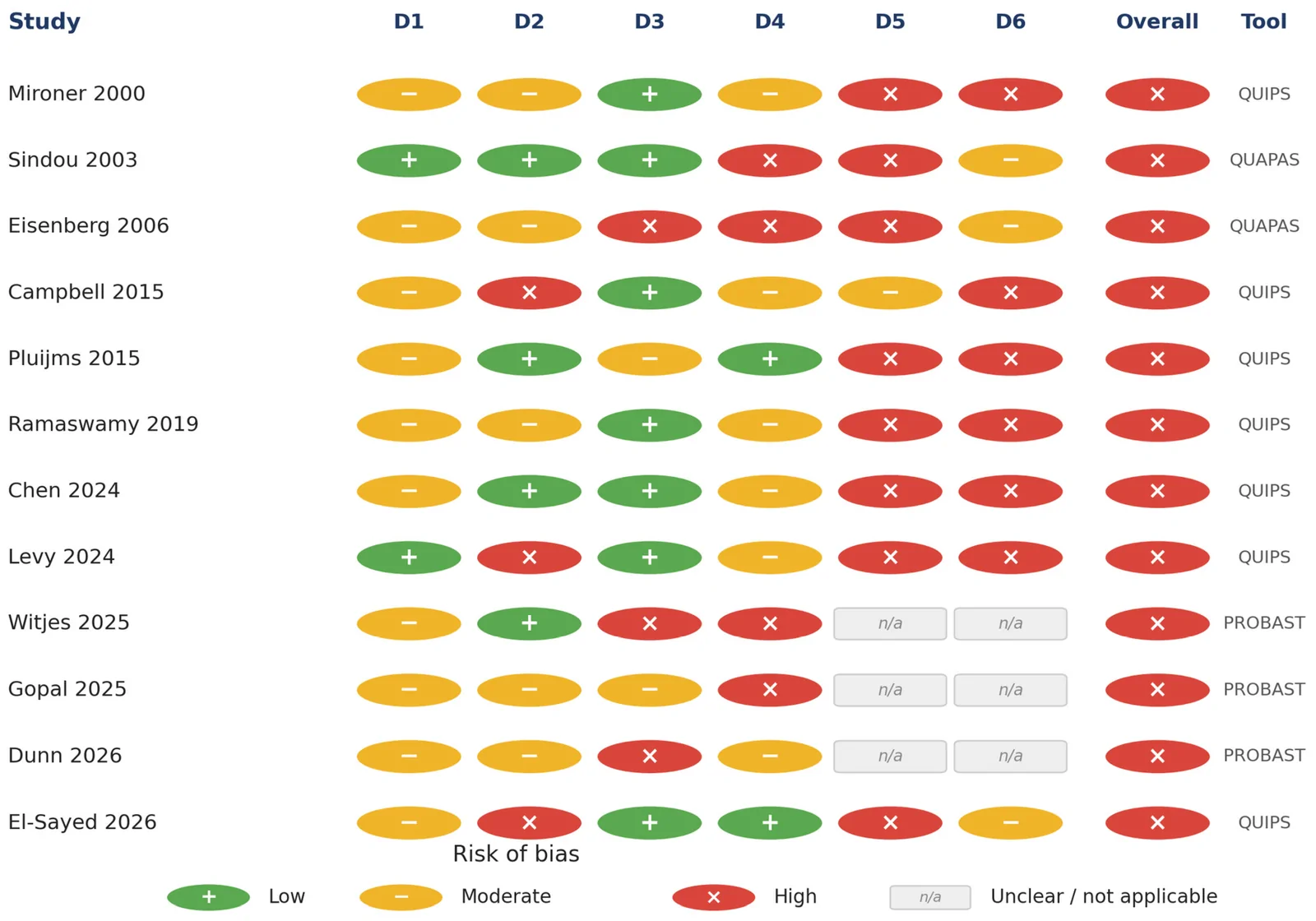
Risk-of-bias summary (consensus of two reviewers) for the 12 included studies [23,24,25,26,27,28,29,30,31,32,33,34]. Domains are harmonized across tools: D1, participants/participation; D2, attrition/predictors; D3, biomarker/predictor measurement; D4, outcome; D5, confounding; D6, analysis/reporting. QUIPS uses six domains; PROBAST and QUAPAS map to D1–D4 (D5–D6 not applicable). Each cell shows the domain-level judgment as a symbol and color: +, low (green); −, moderate (yellow); ×, high risk of bias (red); n/a, not applicable (gray). The overall rating is the highest risk assigned to any applicable domain.

### 2.8. Deviations from the Registered Protocol

The review was conducted as registered, with the following deviations, none of which altered the review question or eligibility criteria: (1) APA PsycINFO was added to strengthen coverage of the psychophysical literature, whereas Google Scholar—listed at registration—was not searched as a standalone source because it does not support reproducible, exportable structured queries and was instead used for forward citation searching. (2) Investigators were not contacted for missing data owing to time constraints. (3) The pre-specified meta-analysis and subgroup/sensitivity analyses were not feasible for the reasons given in Section 2.7; synthesis was restricted to a structured narrative approach. (4) Planned funnel plots and Egger’s tests were not performed because no synthesis reached the ten-study threshold. (5) Formal Grading of Recommendations Assessment, Development and Evaluation (GRADE) summary-of-findings tables were not generated; certainty was judged narratively to be low to very low. (6) Secondary outcomes (function, quality of life, opioid use, psychological measures) were seldom reported within a predictive framework and could not be synthesized. (7) The registration field indicating that only unpublished studies would be sought reflects a data-entry selection on the PROSPERO form; both published and unpublished sources were in fact searched.

## 3. Results

### 3.1. Study Identification and ScreeningLiterature Search

The searches yielded 9383 records. After removal of 4632 duplicates, 4751 records were screened at title and abstract, of which 4727 were excluded. Twenty-four reports were assessed at full text. Twelve were excluded, with reasons: nine were non-peer-reviewed conference abstracts (without a peer-reviewed full report or with insufficient methodological detail), one was a registry protocol reporting no results, and two reported only the effect of SCS on a biomarker rather than the prediction of response (Supplementary File S2). Twelve studies met eligibility and were included; all are peer-reviewed full reports (Figure 2). One conference abstract assessed at full text was superseded by its peer-reviewed full publication, identified through citation searching, which was included in its place so that each cohort is counted once; consequently, each included study is a single peer-reviewed report of a distinct cohort.

### 3.2. Study Characteristics

The 12 included studies were published between 2000 and 2026 and conducted in Europe, North America, and Asia (Table 1). All were small: analyzed sample sizes ranged from 12 to 180 (median approximately 22), and most were single-center prospective pilot cohorts. Populations were predominantly FBSS/radicular neuropathic pain, with individual studies in painful diabetic polyneuropathy, CRPS, and mixed neuropathic pain. Responder definitions varied—most commonly ≥50% pain reduction, but ranged from ≥30% reduction to trial-to-implant conversion or a continuous outcome. By measurement timing and inferential role, nine studies were pre-treatment or intraoperative prognostic-factor or responder-discrimination studies, two were multivariable machine learning prediction models (internal validation only), and one (Levy) used ECAP-derived neural dose measured during closed-loop therapy and is reported as a therapy-embedded surrogate-response measure rather than a pre-treatment predictor. Funding sources and conflict-of-interest disclosures for each included study are provided in Table S1.

### 3.3. Biomarker Domains and Predictive Findings

Table 2 summarizes each study’s biomarker modality, measurement timing, inferential role, and principal finding. The number of studies per biomarker domain was: SSEP/evoked potentials, 2; EEG, 3; MEG, 2; ECAP, 1 (surrogate); QST, 4; and CPM, 3 (several studies contributed to more than one domain). We distinguish throughout between prognostic association and responder discrimination (a baseline marker differs between responders and non-responders), multivariable prediction (a model estimating individual response), and surrogate/monitoring measures (a within-therapy read-out).

#### 3.3.1. Somatosensory and Contact-Heat-Evoked Potentials

The strongest single-study prognostic signal came from one 2003 cohort (*n* = 95) in which normal preoperative dorsal-column central conduction time on SSEP was associated with a 75.4% success rate, whereas success was near-zero when conduction was abolished, leading the authors to advise against implantation in the latter group [23]. This finding derives from a single, historical cohort that used laminotomy electrodes and older SCS technology, spanned several neuropathic etiologies, and was not adjusted for confounders; it should therefore be regarded as a promising but unreplicated prognostic signal requiring confirmation with contemporary SCS paradigms rather than as an established predictor. In a separate small cohort (*n* = 15) with painful diabetic polyneuropathy, forearm contact-heat-evoked potential (CHEP) latencies were longer in responders than in non-responders [24].

#### 3.3.2. EEG and MEG Spectral Features

Several small cohorts reported that resting-state EEG or MEG oscillatory features differed between responders and non-responders. Pain relief correlated with resting alpha and gamma activity in one cohort (*n* = 24; r approximately 0.60 and 0.58) [25]; intraoperative EEG spectral features distinguished responders and supported a machine learning classifier (*n* = 20) [26]; and unsupervised clustering of preoperative EEG identified neurophysiological subtypes that differed in patient-reported outcomes (*n* = 16) [27]. Using MEG, one machine learning study classified chronic-pain status from theta power and the alpha power ratio with 76% accuracy and used the model output to quantify SCS effect (*n* = 25 with SCS) [28], and lower baseline alpha power together with CPM was associated with treatment resistance in a further cohort (*n* = 40) [29]. These signals are individually plausible and partly convergent (alpha and gamma features recur), but each rests on a small, single-center sample.

#### 3.3.3. Quantitative Sensory Testing and Conditioned Pain Modulation

Psychophysical measures were promising but directionally inconsistent. Pain-tolerance threshold and vibration/electrical-detection measures correlated with trial success and the implantation decision in two early pilots [30,31]. Baseline dynamic QST and CPM were associated with 3-month pain in a repeated-measures cohort [32]. For CPM specifically, the direction of association was inconsistent: one FBSS cohort found that an efficient baseline CPM was associated with a higher chance of unsuccessful implantation [33], whereas reduced or altered CPM (with low alpha power) was associated with treatment resistance in another [29]—differences plausibly driven by heterogeneous CPM paradigms, pain phenotypes, and outcome definitions.

#### 3.3.4. Evoked Compound Action Potentials (Therapy-Embedded Surrogate)

One pooled analysis of closed-loop, ECAP-controlled SCS (*n* = 180) identified an objective neural-dose regimen (neural-dose accuracy 2.8 microvolts, dose ratio 1.4) associated with a maximal analgesic effect of approximately 79% [34]. Because the ECAP metric is measured during therapy as a real-time feedback signal rather than before implantation, it is a therapy-embedded surrogate-response measure and does not, on its own, constitute a pre-treatment predictor of who will respond.

### 3.4. Prediction-Model Studies

Two of the 12 studies presented multivariable machine learning models intended to predict SCS response, both using EEG or MEG spectral features with demographic or clinical covariates (Table 3). Reported performance was apparent (derived within the development sample using leave-one-out or cross-validation): 76% classification accuracy in the MEG study [28] and 88.2% accuracy with an area under the receiver operating characteristic curve of 0.879 in the intraoperative-EEG study [26]. A third study applied unsupervised clustering for subtyping rather than supervised response prediction [27]. In every case, the number of candidate predictors was large relative to the sample, calibration was not reported, and none of the models underwent external (out-of-sample) validation; reported performance therefore reflects apparent/internal validation only and is likely optimistic.

### 3.5. Risk of Bias

All 12 studies were at high overall risk of bias (Figure 1). The pattern was consistent across the evidence base: biomarker and predictor measurement (harmonized domain D3) was frequently rated low or moderate risk—reflecting the objective nature of neurophysiological and psychophysical testing—whereas the confounding (D5) and analysis/reporting (D6) domains, and the analysis domain of the prediction models, were frequently rated high risk, owing to small samples, little or no adjustment for confounders, many candidate predictors relative to events, and the absence of external validation. No study was at low or moderate overall risk of bias; accordingly, we do not designate any study as least biased, and the overall certainty of this evidence is low to very low.

## 4. Discussion

This systematic review consolidated evidence from 12 peer-reviewed studies on objective neurophysiological and psychophysical biomarkers for predicting SCS response in chronic neuropathic pain. Three observations emerge. First, most of the evidence consists of prognostic associations or post hoc responder discrimination in small cohorts, not prospectively validated prediction; the two formal prediction models were internally validated only. Second, recurrent signals—preoperative SSEP integrity and resting alpha/gamma EEG/MEG features—are biologically plausible and partly convergent, but each rests on a single small study. Third, the whole evidence base is at high risk of bias, so no biomarker is yet ready to guide clinical selection.

These findings carry a clear implication for precision pain medicine. Because chronic neuropathic pain is mechanistically heterogeneous, the persistent variability in SCS outcomes is unlikely to be resolved by device engineering alone; it requires tools that individualize patient selection. A validated neurophysiological or psychophysical signature—ideally integrated with imaging, molecular, and clinical data through machine learning—could in principle identify likely responders before implantation and spare probable non-responders an invasive, resource-intensive procedure. Our synthesis maps which candidates are closest to that goal while making clear how far they remain from it.

### 4.1. What This Review Adds

This review is distinguished from prior syntheses by its exclusive focus on objective neurophysiological and psychophysical biomarkers as predictors of SCS response, evaluated across every relevant modality and including multivariable and machine learning models. The most recent systematic review of SCS predictors examined patient-level clinical and demographic factors over 2012–2024 and found no strong evidence linking studied factors to outcome, with conflicting results and low-to-very-low certainty across 40 studies [35]. Earlier reviews concentrated on clinical or psychological predictors and trial-based selection [6,36], or on a single biomarker domain such as QST [37]. By contrast, we separated prediction from mechanism—excluding studies reporting only the effect of SCS on a biomarker—and appraised prediction-model studies with design-appropriate tools (PROBAST, QUAPAS), complementing rather than duplicating the existing clinical-predictor literature.

### 4.2. Evidence Gaps

Several recurring limitations currently preclude clinical adoption.

The principal gaps are:

- Small, underpowered, single-center pilot cohorts—typically 12–40 patients—too small for stable multivariable modeling and prone to overfitting [26,29,38,39,40];
- Absence of external and prospective validation. Machine learning models report high apparent internal performance (an area under the receiver operating characteristic curve (AUC) of approximately 0.71–0.91 across the wider literature) but are not externally validated, and proposed predictive frameworks remain unvalidated [41,42,43];
- Methodological heterogeneity. QST and other psychophysical protocols are inconsistent, and few apply the full validated DFNS battery, limiting comparability and pooling [37];
- Non-standardized outcome definitions (30% vs. 50% vs. trial-to-implant conversion; trial vs. 3–12-month outcomes), undermining cross-study comparison [29,39];
- Conflicting, non-replicated psychophysical signals, particularly for CPM [37,38];
- Uncertain generalizability of legacy neurophysiology (e.g., SSEP) to modern high-frequency, burst, and closed-loop paradigms [23,44], and persistent reliance on the invasive trial because no objective biomarker yet reliably identifies responders [6,36].

### 4.3. Future Directions

Directions most likely to advance the field include:

- Large prospective cohorts and registries with individual-patient data [35];
- Multimodal biomarker integration (neurophysiologic, imaging, psychophysical, molecular), which outperforms single markers, e.g., radiomics plus clinical data reaching AUC approximately 0.91 [39,40,42];
- Machine learning with rigorous, prospective external validation, standardization, and attention to regulatory issues [41,45];
- Standardized psychophysical protocols (validated QST battery) to enable comparability and meta-analysis [37];
- Objective neurophysiologic candidates for pre-implant selection, including resting-state functional connectivity and alpha oscillatory power [29,39];
- Extending closed-loop ECAP feedback from a therapy-embedded surrogate toward validated predictive and monitoring biomarkers [34,46], with consensus responder definitions to enable pooling and replication.

### 4.4. Standardizing Objective Neurophysiologic Biomarker Acquisition

A major barrier to translating and adopting neurophysiologic biomarkers into clinical neuromodulation is their sensitivity to acquisition and analytic conditions. For evoked potentials and resting-state EEG and MEG, measured features can vary with electrode type and montage, inter-electrode distance, skin preparation and impedance, reference and filter settings, stimulus parameters, artifact rejection, and spectral analysis methods [47]. Consequently, a numerical threshold derived at one center may not reproduce when different equipment or processing pipelines are used elsewhere. This technical variability is particularly important in a literature already characterized by small samples and limited external validation and may partly explain the inconsistent findings across EEG, MEG, and psychophysical studies. 

Several measures could improve reproducibility and move the field toward standardized neurophysiologic biomarker assessment. First, when biologically appropriate, robust categorical readouts may be more transferable than highly calibration-dependent continuous thresholds. For example, classifying dorsal-column conduction as preserved versus abolished using somatosensory evoked potentials (SSEPs)—the form in which the strongest association in this review was reported [23]—may be less sensitive to inter-site calibration than relying on a specific latency threshold. This approach is also consistent with international recommendations for the clinical application of SSEPs [48].

Second, biomarker protocols should incorporate within-subject comparisons and appropriate normative reference data whenever possible. Side-to-side comparisons with an unaffected homologous nerve or dermatome can reduce interindividual variability. Similarly, comparison with validated healthy-control datasets or published normative values can improve interpretation across sites and devices. These approaches are already established in clinical electrodiagnosis and provide a practical framework for biomarker studies [48].

Third, acquisition and analysis should be harmonized across studies. Pre-specified acquisition parameters, preregistered analysis pipelines, and common data structures would allow candidate features to be calculated more consistently and facilitate multicenter pooling and external validation. Existing frameworks provide a foundation for this approach, including the Organization for Human Brain Mapping (OHBM) Committee on Best Practices in Data Analysis and Sharing (COBIDAS) recommendations for magnetoencephalography and electroencephalography (MEEG) [47] and the Brain Imaging Data Structure extension for EEG (EEG-BIDS) [49].

Psychophysical biomarkers require similar standardization. Rather than relying on locally developed quantitative sensory testing protocols, studies should preferentially use validated frameworks such as the German Research Network on Neuropathic Pain (DFNS) quantitative sensory testing battery and its published reference values [50], consistent with guidance from the Neuropathic Pain Special Interest Group (NeuPSIG) [51]. Conditioned pain modulation (CPM) protocols should likewise follow standardized procedures for stimulus selection, timing, and outcome calculation [52]. Importantly, candidate biomarkers should be accompanied by estimates of test–retest reliability and minimal detectable change whenever these are available. Without these measures, it remains difficult to determine whether longitudinal differences represent biological change or measurement variability.

Achieving this level of standardization will likely require coordination beyond individual laboratories. Relevant professional organizations include the International Federation of Clinical Neurophysiology and the American Clinical Neurophysiology Society for evoked-potential and EEG/MEG methodology [48], and the DFNS and NeuPSIG of the International Association for the Study of Pain for psychophysical testing [50,51]. The International Neuromodulation Society and North American Neuromodulation Society could then help translate these standards into neuromodulation research and clinical practice, including through consensus initiatives such as the Neuromodulation Appropriateness Consensus Committee [3].

A multicenter standardization effort modeled on the DFNS approach—including consensus acquisition protocols, shared reference values, and standardized reporting—could provide a common measurement framework for neuromodulation biomarker research [50]. Pairing this framework with Initiative on Methods, Measurement, and Pain Assessment in Clinical Trials (IMMPACT)-aligned definitions of response and clinical outcomes [53] would further improve comparability across studies. Journals, registries, and funding agencies could accelerate adoption by incorporating these standards into reporting requirements and prospective study design.

### 4.5. Strengths and Limitations

Strengths include prospective registration, a comprehensive multi-database and registry search with librarian PRESS peer-review, dual independent screening and extraction with quantified agreement, restriction to peer-reviewed reports, and design-appropriate risk-of-bias appraisal. Limitations arise mainly from the evidence base: the included studies were few, small, clinically and methodologically heterogeneous, and uniformly at high risk of bias, precluding meta-analysis. We did not contact authors for missing data; we could not formally assess reporting bias (funnel plots) or generate GRADE summary-of-findings tables given the sparse, non-poolable evidence; and two recent EEG cohorts from the same center may share participants, which we flag as a potential source of non-independence. Restricting inclusion to peer-reviewed reports excludes recent conference material, trading breadth for verifiability and methodological transparency.

## 5. Conclusions

Objective neurophysiological and psychophysical biomarkers—most notably the preoperative SSEP and resting-state EEG and MEG spectral features—show promise for predicting SCS treatment response in chronic neuropathic pain. The current evidence, however, is limited, heterogeneous, small, at high risk of bias, and largely confined to prognostic associations and internally validated models. Adequately powered, prospectively designed, multimodal studies with standardized biomarker acquisition, pre-specified responder definitions, and external validation of prediction models are needed before any biomarker can support precision patient selection and reduce reliance on the invasive screening trial [35,43,45].

## Figures and Tables

**Figure 2:**
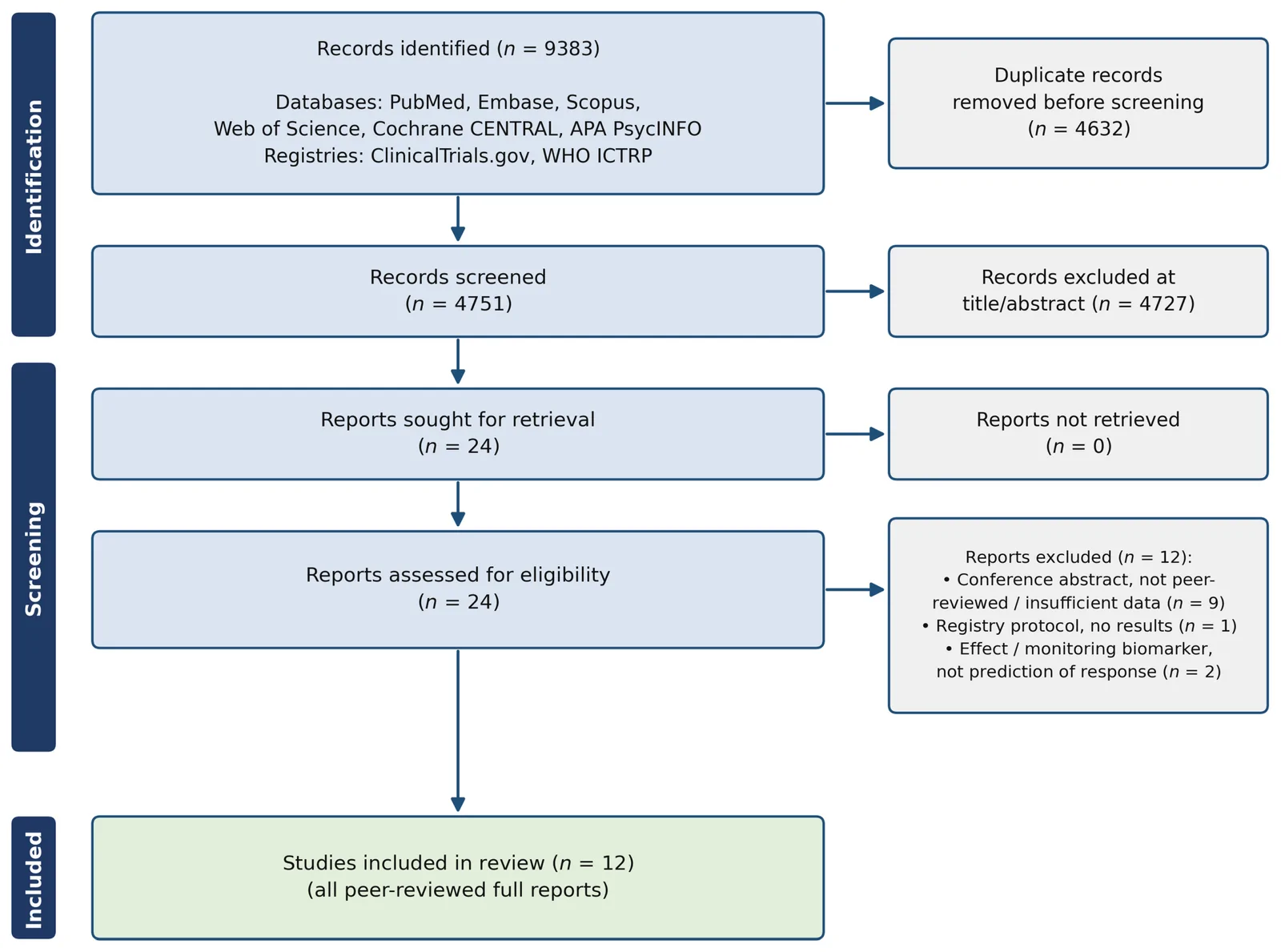
PRISMA 2020 flow diagram of study identification, screening, and inclusion. Arrows indicate the flow of records through the review; blue boxes denote records progressing through identification, screening, and eligibility; gray boxes denote records removed or excluded (with reasons); and the green box denotes the studies included. SCS, spinal cord stimulation.

**Table 1:** Characteristics of the 12 included studies.

First Author (Year)	Country	Design	N Analyzed	Population/Etiology	SCS Paradigm	Responder Definition	Overall RoB
Mironer et al. (2000) [30]	USA	Prospective pilot, before–after	44 (+9 controls)	Chronic pain, SCS-trial candidates	SCS trial	Trial success → permanent-implant success	High
Sindou et al. (2003) [23]	France	Prospective cohort	95	Neuropathic (peripheral nerve, radicular, root avulsion, cord)	SCS, laminotomy electrodes	≥50% long-term pain relief	High
Eisenberg et al. (2006) [31]	USA/Israe	Prospective pilot	12	FBSS radicular leg pain or CRPS	SCS trial	Permanent-implant decision	High
Campbell et al. (2015) [32]	USA	Repeated-measures observational pilot	24	Chronic pain, mixed	SCS trial → permanent	3-mo clinical pain (continuous)	High
Pluijms et al. (2015) [24]	Netherlands	Prospective pilot cohort	15	Painful diabetic polyneuropathy	SCS trial → permanent	Clinically relevant relief at 12 mo	High
Ramaswamy et al. (2019) [33]	UK	Single-center prospective pilot	23 (19 at 3 mo)	FBSS, unilateral lumbar radiculopathy	SCS	Successful implant at 3 mo	High
Chen et al. (2024) [25]	China	Prospective cohort	24	Neuropathic pain	Short-term SCS implantation	Analgesia responder (VAS reduction)	High
Levy et al. (2024) [34]	Multinational	Pooled analysis of 3 studies	180	Chronic back and leg pain	ECAP closed-loop SCS	Maximal analgesic effect (≤3 mo)	High
Witjes et al. (2025) [28]	Netherlands	Case-control + ML	75 (25 SCS)	Chronic pain; pain-free controls	Tonic/burst/sham	Pain classification; SCS-effect quantification	High
Gopal et al. (2025) [26]	USA	Prospective cohort + ML	20	Chronic back/leg pain	Tonic and high-frequency	Responder (composite at 3 mo)	High
Dunn et al. (2026) [27]	USA	Prospective cohort + unsupervised ML	16	Chronic pain scheduled for SCS	SCS (pre-implant)	PRO differences across EEG clusters	High
El-Sayed et al. (2026) [29]	Canada	Prospective cohort (case–control)	40 (+29 HC)	Neuropathic pain	12-day SCS trial	≥30% pain reduction post-trial	High

Abbreviations: CRPS, complex regional pain syndrome; ECAP, evoked compound action potential; EEG, electroencephalography; FBSS, failed back surgery syndrome; HC, healthy controls; ML, machine learning; PRO, patient-reported outcome; RoB, risk of bias; SCS, spinal cord stimulation; VAS, visual analogue scale.

**Table 2:** Biomarker modality, timing, inferential role, and principal findings.

First Author (Year)	Biomarker (Modality)	Timing	Inferential Role	Key Parameter	Principal Finding	Discrimination (Apparent)
Sindou et al. (2003) [23]	SSEP	Pre-treatment	Prognostic factor	Central conduction time (normal vs. abnormal)	Normal SSEP → 75.4% success; abnormal → near-0%	NR (qualitative)
Pluijms et al. (2015) [24]	CHEP (evoked potential)	Pre-treatment + 2-wk trial	Responder discrimination	Forearm CHEP N2/P2 latencies	Longer latencies in responders (volar N2, *p* < 0.01)	NR
Mironer et al. (2000) [30]	QST	Before/after trial	Prognostic factor	Pain-tolerance threshold (PTT)	PTT correlated with trial and implant success	NR
Eisenberg et al. (2006) [31]	QST	Pre + 7-day trial	Prognostic factor	Vibration threshold; electrical tolerance 5/250 Hz	Correlated with the implantation decision	Sens/Spec reported (e.g., vibration 100%/50%)
Campbell et al. (2015) [32]	QST/CPM (dynamic)	Baseline	Prognostic factor	Central sensitization; CPM	Baseline CS and CPM associated with 3-mo pain (adjusted)	NR
Ramaswamy et al. (2019) [33]	QST/CPM	Baseline	Prognostic factor	CPM efficiency	Efficient baseline CPM → higher chance of unsuccessful implant	NR
Chen et al. (2024) [25]	Resting EEG	During short-term implant	Responder discrimination	Alpha and gamma oscillatory power	Pain relief ~ alpha (r = 0.60) and gamma (r = 0.58)	NR
El-Sayed et al. (2026) [29]	MEG (alpha) + CPM	Pre-treatment	Prognostic factor	Baseline alpha power; CPM	Lower alpha with altered CPM → treatment resistance	NR
Witjes et al. (2025) [28]	MEG	During stimulation (resting)	Prediction model (classification)	Theta power; alpha power ratio	Classified chronic-pain status; quantified SCS effect	76% accuracy (internal)
Gopal et al. (2025) [26]	Intraoperative EEG	Intraoperative	Prediction model	EEG spectral features + clinical	Higher alpha–theta ratio in responders	88.2% accuracy; AUC 0.879 (internal)
Dunn et al. (2026) [27]	Preoperative EEG	Pre-treatment	Exploratory subtyping (unsupervised)	EEG spectral clusters (alpha/beta/theta, entropy)	Three clusters differing in PROs	NR (unsupervised)
Levy et al. (2024) [34]	ECAP	During therapy (closed-loop)	Therapy-embedded surrogate	Neural-dose accuracy (2.8 µV), dose ratio (1.4)	Neural-dose regimen~79% maximal analgesic effect	NR

Abbreviations: AUC, area under the receiver operating characteristic curve; CHEP, contact heat evoked potential; CPM, conditioned pain modulation; CS, central sensitization; ECAP, evoked compound action potential; EEG, electroencephalography; MEG, magnetoencephalography; PTT, pain-tolerance threshold; QST, quantitative sensory testing; SCS, spinal cord stimulation; SSEP, somatosensory evoked potential. NR, not reported.

**Table 3:** Multivariable prediction-model studies (CHARMS/PROBAST descriptors).

First Author (Year)	Model Type	Predictors/Selection	Sample (EPV)	Internal Validation	External Validation	Reported Performance (Apparent)
Witjes et al. (2025) [28]	Automated ML (classification/regression)	MEG spectral features (theta power, alpha ratio)	*n* = 75 (25 SCS); low EPV	Cross-validation	None	76% classification accuracy
Gopal et al. (2025) [26]	ML classifier (decision tree; PCA + forward selection)	Intraoperative EEG spectral features + demographics	*n* = 20; very low EPV	Leave-one-out CV	None	88.2% accuracy; AUC 0.879; F1 0.857
Dunn et al. (2026) [27]	Unsupervised k-means clustering	Preoperative EEG spectral features	*n* = 16	n/a (unsupervised)	None	Cluster–PRO associations (no responder AUC)

Abbreviations: AUC, area under the receiver operating characteristic curve; CV, cross-validation; EEG, electroencephalography; EPV, events per variable; MEG, magnetoencephalography; ML, machine learning; PRO, patient-reported outcome; SCS, spinal cord stimulation. n/a, not applicable; None, explicitly reported as absent.

## Data Availability

All data supporting this review are contained within the article, its tables and Supplementary Files, and the registered protocol (PROSPERO CRD420261410302).

## Supplementary Materials

The following supporting information can be downloaded at: https://www.mdpi.com/article/10.3390/jpm16100486/s1, Supplementary File S1, complete database-specific electronic search strategies for all six databases and two registries, with the final search date and per-source record yields; Supplementary File S2, list of reports excluded at full-text screening with reasons; Supplementary File S3, PRISMA 2020 Checklist [15]. Table S1, full funding and conflict-of-interest disclosures for each included study.
