# Supplementary material for: Neurophysiological and Psychophysical Biomarkers for Predicting Spinal Cord Stimulation Treatment Response in Chronic Neuropathic Pain: A Systematic Review
Source: J Pers Med. 2026 Sep 22;16(10):486. doi: 10.3390/jpm16100486 (PMC13706508; doi:10.3390/jpm16100486)
Supplement: Supplementary file 1 [file jpm-16-00486-s001.zip › Supplementary File S1. complete database-specific electronic search strategies for all six databases and two registries, with the final search date and per-source record yields.pdf]

Supplementary File S1. complete database-specific electronic search strategies for all six databases and two registries, with the final search date and per-source record yields

Neurophysiological and Psychophysical Biomarkers for Predicting Spinal Cord Stimulation Treatment Response in Chronic Neuropathic Pain: A Systematic Review · PROSPERO CRD420261410302

All databases and registries were searched on 4 June 2026, with no language or date restrictions. The strategy combines three concept blocks. The primary search = Concept 1 AND Concept 2 AND Concept 3; a sensitive supplementary search = Concept 1 AND Concept 2. Records retrieved per source (primary / supplementary): PubMed 347/2,046; Embase 580/2,776; Scopus 673/2,349; Web of Science 413/2,092; Cochrane CENTRAL 78/383; APA PsycINFO 106/466; ClinicalTrials.gov 125; WHO ICTRP 30. After de-duplication in Rayyan, 4,751 unique records were screened.

The electronic search strategy was developed in collaboration with a medical research librarian (Melissa Funaro, MLS; Harvey Cushing/John Hay Whitney Medical Library, Yale University) and peer-reviewed against the PRESS 2015 checklist prior to execution.

Record yields per source (searches executed 4 June 2026)

The table reports records retrieved per database for the sensitive screening set (Concept 1 AND Concept 2). "Pre-exclusion" is the raw retrieval; "Refined (final)" is the set exported for screening after the title-only exclusion filter. The six databases yielded 9,383 records across nine export files—the number imported into Rayyan for de-duplication and screening, consistent with the PRISMA 2020 flow diagram (Figure 1) in the main text.

Database	Pre-exclusion	Refined (final)	Export
PubMed / MEDLINE	2,046	1,902	.nbib (1 file)
Embase (Ovid)	2,776	2,531	RIS (2 files)
Scopus	2,349	2,164	RIS (1 file)
Web of Science	2,092	1,937	RIS (2 files)
APA PsycINFO (Ovid)	466	471	RIS (1 file)
Cochrane CENTRAL	384	378	RIS (377 trials + 1 review)
**TOTAL (pre-deduplication)**	**10,113**	**9,383**	**9 files**

PsycINFO note: the refined count (471) is marginally above the pre-exclusion count (466) because the title-only exclusion was not applied on the final PsycINFO run; it removes effectively nothing in this database, so the set is accepted as-is.

Trial registries: ClinicalTrials.gov and the WHO International Clinical Trials Registry Platform (Section 7) were additionally searched and screened. Their records are reported separately and are not part of the 9,383 database records above; they contributed no additional included studies.

De-duplication and screening

The 9,383 records (nine files, six databases) were imported into Rayyan. Manual matching removed 4,584 duplicates (4,103 by DOI and 481 by exact title), giving an upper-bound unique estimate of 4,799. This is an upper bound: 157 of the 377 Cochrane CENTRAL trials carry no DOI and match by title only, and CENTRAL title formatting (en-dashes, embedded page ranges) differs from PubMed/Embase, so simple matching under-merges them. Rayyan's fuzzy de-duplication at import merged additional near-duplicates, yielding 4,632 duplicates removed and 4,751 unique records taken to title/abstract screening. Of these, 4,727 were excluded at title/abstract, 24 reports were assessed at full text, 12 were excluded (Supplementary File S2), and 12 studies were included (Figure 1).

Unique contribution by source (upper bound; de-duplication order PubMed first)

Source	Unique records (upper bound)
PubMed	1,895
Embase	1,279
Web of Science	678
Scopus	597
Cochrane CENTRAL	297 (inflated — see note)
APA PsycINFO	53
**TOTAL (upper bound)**	**4,799**

Cochrane CENTRAL note: its 297 apparent unique records are inflated. Most are true duplicates already captured via PubMed/Embase but are under-merged by exact matching because many CENTRAL trials lack DOIs and use divergent title formatting. The authoritative unique pool after Rayyan fuzzy de-duplication is therefore lower than 4,799 (the 4,751 records screened).

1. PubMed / MEDLINE (NCBI)

Concept 1 — Spinal cord stimulation

```
("Spinal Cord Stimulation"[MeSH] OR "spinal cord stimulat*"[tiab] OR SCS[tiab] OR "dorsal column
stimulat*"[tiab] OR "epidural stimulat*"[tiab] OR "closed-loop stimulat*"[tiab] OR "ECAP-
controlled"[tiab]
OR "evoked compound action potential*"[tiab] OR ECAP[tiab] OR "burst stimulat*"[tiab] OR
BurstDR[tiab] OR
"high-frequency stimulat*"[tiab] OR HF10[tiab] OR "10 kHz"[tiab] OR "tonic stimulat*"[tiab] OR
"differential target multiplexed"[tiab] OR DTMP[tiab])
```

Concept 2 — Neurophysiological & psychophysical biomarkers

```
("Electroencephalography"[MeSH] OR electroencephalograph*[tiab] OR EEG[tiab] OR
"Magnetoencephalography"[MeSH]
OR magnetoencephalograph*[tiab] OR MEG[tiab] OR "Evoked Potentials, Somatosensory"[MeSH] OR
"somatosensory
evoked potential*"[tiab] OR SSEP[tiab] OR "central conduction time"[tiab] OR "conditioned pain
modulation"[tiab] OR "diffuse noxious inhibitory control"[tiab] OR DNIC[tiab] OR "quantitative
sensory
testing"[tiab] OR QST[tiab] OR "temporal summation"[tiab] OR wind-up[tiab] OR windup[tiab] OR
"dynamic
mechanical allodynia"[tiab] OR "pressure pain threshold"[tiab] OR "thermal threshold"[tiab] OR
"mechanical
threshold"[tiab] OR "pain threshold"[tiab] OR "pain tolerance"[tiab] OR "brain oscillat*"[tiab] OR
"alpha
power"[tiab] OR "theta power"[tiab] OR "beta power"[tiab] OR "gamma power"[tiab] OR "peak
frequency"[tiab]
OR "cortical excitab*"[tiab] OR "thalamocortical dysrhythmia"[tiab] OR "neural signature*"[tiab] OR
psychophysic*[tiab])
```

Concept 3 — Prediction / prognosis

```
(predict*[tiab] OR prognos*[tiab] OR predictor*[tiab] OR "treatment response"[tiab] OR "treatment
outcome"[tiab] OR responder*[tiab] OR nonresponder*[tiab] OR non-responder*[tiab] OR "patient
selection"[tiab] OR "outcome predict*"[tiab] OR "diagnostic accuracy"[tiab] OR "prognostic
accuracy"[tiab]
OR "area under the curve"[tiab] OR "machine learning"[tiab] OR "artificial intelligence"[tiab] OR
discriminat*[tiab])
```

Combined: (Concept 1) AND (Concept 2) AND (Concept 3) for the primary search; (Concept 1) AND (Concept 2) for the supplementary search. English-language filter applied at the final step.

2. Embase (Ovid, Emtree)

```
1   exp spinal cord stimulation/
2   (spinal cord stimulat* or SCS or dorsal column stimulat* or epidural stimulat* or closed-loop
    stimulat* or ECAP-controlled or evoked compound action potential* or ECAP or burst stimulat* or
    BurstDR or high-frequency stimulat* or HF10 or 10 kHz or tonic stimulat* or differential target
```

```
    multiplexed or DTMP or neuromodulat*).ti,ab,kw.
3   1 or 2
4   exp electroencephalography/ or exp magnetoencephalography/ or exp somatosensory evoked 
potential/
    or exp quantitative sensory testing/
5   (electroencephalograph* or EEG or magnetoencephalograph* or MEG or somatosensory evoked 
potential*
    or SSEP or SEP or central conduction time or conditioned pain modulation or CPM or diffuse 
noxious
    inhibitory control or DNIC or quantitative sensory testing or QST or temporal summation or wind-
up
    or windup or dynamic mechanical allodynia or pressure pain threshold or PPT or thermal threshold 
or
    mechanical threshold or pain threshold or pain tolerance or neurophysiolog* or electrophysiolog* 
or
    brain oscillat* or spectral analysis or alpha power or theta power or beta power or gamma power 
or
    peak frequency or cortical excitab* or thalamocortical dysrhythmia or resting state or 
biomarker* or
    neural marker* or neural signature* or psychophysic*).ti,ab,kw.
6   4 or 5
7   (predict* or prognos* or predictor* or treatment response or treatment outcome or responder* or
    nonresponder* or non-responder* or patient selection or diagnostic accuracy or prognostic 
accuracy
    or area under the curve or AUC or machine learning or artificial intelligence or classification 
or
    discriminat*).ti,ab,kw.
8   3 and 6 and 7        <- primary
9   3 and 6              <- supplementary
```

3. Cochrane CENTRAL (Cochrane Library)

```
#1   MeSH descriptor: [Spinal Cord Stimulation] explode all trees
#2   (spinal cord stimulat* or SCS or dorsal column stimulat* or epidural stimulat* or ECAP* or 
burst
     stimulat* or BurstDR or high-frequency stimulat* or HF10 or "10 kHz" or tonic stimulat* or
     differential target multiplexed or DTMP or neuromodulat*):ti,ab,kw
#3   #1 or #2
#4   MeSH descriptor: [Electroencephalography] explode all trees
#5   MeSH descriptor: [Magnetoencephalography] explode all trees
#6   MeSH descriptor: [Evoked Potentials, Somatosensory] explode all trees
#7   (electroencephalograph* or EEG or magnetoencephalograph* or MEG or "somatosensory evoked
     potential*" or SSEP or SEP or "central conduction time" or "conditioned pain modulation" or CPM 
or
     DNIC or "quantitative sensory testing" or QST or "temporal summation" or wind-up or windup or
     "dynamic mechanical allodynia" or "pressure pain threshold" or PPT or "thermal threshold" or
     "mechanical threshold" or "pain threshold" or "pain tolerance" or neurophysiolog* or
     electrophysiolog* or "spectral analysis" or "alpha power" or "resting state" or biomarker* or
     "neural signature*" or psychophysic*):ti,ab,kw
#8   #4 or #5 or #6 or #7
#9   (predict* or prognos* or predictor* or "treatment response" or responder* or "patient 
selection"
     or "diagnostic accuracy" or "machine learning" or discriminat*):ti,ab,kw
#10  #3 and #8 and #9
```

4. Scopus (Advanced search, TITLE-ABS-KEY)

```
( TITLE-ABS-KEY("spinal cord stimulat*" OR SCS OR "dorsal column stimulat*" OR "epidural stimulat*" 
OR
  "closed-loop stimulat*" OR "ECAP-controlled" OR "evoked compound action potential*" OR ECAP OR
```

```
"burst
  stimulat*" OR BurstDR OR "high-frequency stimulat*" OR HF10 OR "10 kHz" OR "tonic stimulat*" OR
  "differential target multiplexed" OR DTMP OR neuromodulat*) )
AND
( TITLE-ABS-KEY(electroencephalograph* OR EEG OR magnetoencephalograph* OR MEG OR "somatosensory
evoked
  potential*" OR SSEP OR SEP OR "central conduction time" OR "conditioned pain modulation" OR CPM OR
  "diffuse noxious inhibitory control" OR DNIC OR "quantitative sensory testing" OR QST OR "temporal
  summation" OR wind-up OR windup OR "dynamic mechanical allodynia" OR "pressure pain threshold" OR
PPT
  OR "thermal threshold" OR "mechanical threshold" OR "pain threshold" OR "pain tolerance" OR
  neurophysiolog* OR electrophysiolog* OR "brain oscillat*" OR "spectral analysis" OR "alpha power"
OR
  "theta power" OR "beta power" OR "gamma power" OR "peak frequency" OR "cortical excitab*" OR
  "thalamocortical dysrhythmia" OR "resting state" OR biomarker* OR "neural marker*" OR "neural
  signature*" OR psychophysic*) )
AND
( TITLE-ABS-KEY(predict* OR prognos* OR predictor* OR "treatment response" OR "treatment outcome" OR
  responder* OR nonresponder* OR "non-responder*" OR "patient selection" OR "diagnostic accuracy" OR
  "prognostic accuracy" OR "area under the curve" OR AUC OR "machine learning" OR "artificial
  intelligence" OR classification OR discriminat*) )
```

5. Web of Science Core Collection (TS = Topic)

```
TS=("spinal cord stimulat*" OR SCS OR "dorsal column stimulat*" OR "epidural stimulat*" OR "closed-
loop
stimulat*" OR "ECAP-controlled" OR "evoked compound action potential*" OR ECAP OR "burst stimulat*"
OR
BurstDR OR "high-frequency stimulat*" OR HF10 OR "10 kHz" OR "tonic stimulat*" OR "differential
target
multiplexed" OR DTMP OR neuromodulat*)
AND
TS=(electroencephalograph* OR EEG OR magnetoencephalograph* OR MEG OR "somatosensory evoked
potential*"
OR SSEP OR SEP OR "central conduction time" OR "conditioned pain modulation" OR CPM OR "diffuse
noxious
inhibitory control" OR DNIC OR "quantitative sensory testing" OR QST OR "temporal summation" OR
wind-up
OR windup OR "dynamic mechanical allodynia" OR "pressure pain threshold" OR PPT OR "thermal
threshold"
OR "mechanical threshold" OR "pain threshold" OR "pain tolerance" OR neurophysiolog* OR
electrophysiolog*
OR "brain oscillat*" OR "spectral analysis" OR "alpha power" OR "theta power" OR "beta power" OR
"gamma
power" OR "peak frequency" OR "cortical excitab*" OR "thalamocortical dysrhythmia" OR "resting
state" OR
biomarker* OR "neural marker*" OR "neural signature*" OR psychophysic*)
AND
TS=(predict* OR prognos* OR predictor* OR "treatment response" OR "treatment outcome" OR responder*
OR
nonresponder* OR "non-responder*" OR "patient selection" OR "diagnostic accuracy" OR "prognostic
accuracy" OR "area under the curve" OR AUC OR "machine learning" OR "artificial intelligence" OR
classification OR discriminat*)
```

6. APA PsycINFO (Ovid)

```
1   (spinal cord stimulat* or SCS or dorsal column stimulat* or epidural stimulat* or neuromodulat*
or
    evoked compound action potential* or ECAP or burst stimulat* or high-frequency stimulat* or HF10
or
```

```
    10 kHz or tonic stimulat* or differential target multiplexed or DTMP).ti,ab,id.
2   (electroencephalograph* or EEG or magnetoencephalograph* or MEG or somatosensory evoked
potential*
    or SSEP or central conduction time or conditioned pain modulation or CPM or diffuse noxious
    inhibitory control or DNIC or quantitative sensory testing or QST or temporal summation or wind-
up
    or windup or dynamic mechanical allodynia or pressure pain threshold or thermal threshold or
    mechanical threshold or pain threshold or pain tolerance or neurophysiolog* or electrophysiolog*
or
    brain oscillat* or spectral analysis or alpha power or theta power or beta power or gamma power
or
    peak frequency or cortical excitab* or thalamocortical dysrhythmia or resting state or
biomarker* or
    neural signature* or psychophysic*).ti,ab,id.
3   (predict* or prognos* or predictor* or treatment response or treatment outcome or responder* or
    nonresponder* or non-responder* or patient selection or diagnostic accuracy or prognostic
accuracy
    or area under the curve or AUC or machine learning or artificial intelligence or classification
or
    discriminat*).ti,ab,id.
4   1 and 2 and 3
```

7. Trial registries

ClinicalTrials.gov (Advanced Search → Other terms) and WHO ICTRP (basic search):

```
spinal cord stimulation AND (EEG OR "evoked potential" OR "quantitative sensory testing" OR
"conditioned pain modulation" OR biomarker OR predict*)
```

8. Supplementary searching

- Backward citation: reference lists of all included studies and of relevant existing reviews were screened.
- Forward citation: “cited by” searches were run on each included study in Scopus/Google Scholar.
- Grey literature: conference abstracts (INS, NANS, IASP; last 5 years) and preprints (medRxiv, bioRxiv) were identified and screened but were not eligible for inclusion, as the review was restricted to peer-reviewed reports (see manuscript Section 2.2 and the Section 2.8 deviations).
- Corresponding authors of included studies were not contacted for unpublished or in-press data owing to time constraints (manuscript Section 2.8).
