# Supplementary material for: Neurophysiological and Psychophysical Biomarkers for Predicting Spinal Cord Stimulation Treatment Response in Chronic Neuropathic Pain: A Systematic Review
Source: J Pers Med. 2026 Sep 22;16(10):486. doi: 10.3390/jpm16100486 (PMC13706508; doi:10.3390/jpm16100486)
Supplement: Supplementary file 1 [file jpm-16-00486-s001.zip › Supplementary File S2. list of reports excluded at full-text screening with reasons.pdf]

# Supplementary File S2. list of reports excluded at full-text screening with reasons

*Neurophysiological and Psychophysical Biomarkers for Predicting SCS Treatment Response — PROSPERO CRD420261410302*

Twenty-four reports were assessed at full text; 12 were included and 12 were excluded, as listed below. Where a conference abstract and a later full publication described the same cohort, the abstract was excluded and the peer-reviewed full publication was included.

| # | Author(s) | Title | Source / year | Reason for exclusion |
|---|---|---|---|---|
| 1 | Mehta, V.; Ramaswamy, S.; Wodehouse, T. | QST as a biomarker for phenotyping patients undergoing spinal cord stimulation. | Neuromodulation (conference abstract); 2017 | Conference abstract, not peer-reviewed; same cohort as Ramaswamy 2019 (included) |
| 2 | Ramaswamy, S.; Wodehouse, T.; Mehta, V. | Sensory profiling of patients undergoing spinal cord stimulation for neuropathic back and leg pain following FBSS. | British Journal of Pain (conference abstract); 2017 | Conference abstract, not peer-reviewed; same cohort as Ramaswamy 2019 (included) |
| 3 | Steyvers, M.; Wilder-Smith, O.H.; Groen, G.J.; et al. | Conditioned pain modulation as a predictor of pain reduction following SCS in FBSS. | European Journal of Pain Supplements (congress abstract); 2011 | Conference abstract, not peer-reviewed; insufficient methodological detail |
| 4 | See, K.; Ho, R.; Coombes, S.; Fang, R. | TL1 Team Approach to Predicting Response to SCS for Chronic Low Back Pain. | J. Clinical and Translational Science (conference abstract); 2021 | Conference abstract, not peer-reviewed |
| 5 | Gopal, J.; Bao, J.; Paniccioli, S.; et al. | Machine Learning Predicts SCS Surgery Outcomes for Chronic Pain Patients. | Neuromodulation (conference abstract); 2024 | Conference abstract superseded by peer-reviewed full publication (Gopal 2025, Sci. Rep.), which was included |
| 6 | Telkes, I.; Gopal, J.; Dunn, A.; et al. | Explainable Machine Learning Pipeline Predicts SCS Responders (ID# 1907064). | Neuromodulation (conference abstract); 2025 | Conference abstract, not peer-reviewed; same centre/cohort as Dunn 2026 (included) |
| 7 | Telkes, I.; Dunn, A.; Gopal, J.; et al. | EEG Distinctions Between Responders and Non-responders to SCS (ID# 1907423). | Neuromodulation (conference abstract); 2025 | Conference abstract, not peer-reviewed; same centre/cohort as Dunn 2026 (included) |
| 8 | Pridgeon, M. | The role of EEG theta power and SSEPs in SCS baseline responder selection in FBSS (PO 253). | Clinical Neurophysiology (conference abstract); 2026 | Conference abstract, not peer-reviewed |
| 9 | Verrills, P.; Lee, D.; Dawson, C.; Bradley, K. | Correlation between short-term adaptation of paresthesia and pain relief from high-kHz epidural SCS. | Neuromodulation (conference abstract); 2020 | Conference abstract, not peer-reviewed; biomarker measured post-implant (not pre-treatment/intraoperative) |
| 10 | MOPPStim Investigators | Mechanisms and outcome-prognostication for paresthesia-based and -free SCS (MOPPStim). | ClinicalTrials.gov registry protocol; 2018 | Registry protocol reporting no results (awaiting classification) |
| 11 | Russo, M.; Brooker, C.; Cousins, M.J.; et al. | Long-term results from the Avalon study: feedback-controlled SCS using ECAP. | Neuromodulation; 2018 | Reports device clinical outcomes/monitoring, not prediction of response |
| 12 | Kim, S.J.; et al. | Quantitative evaluation of tonic SCS in treating chemotherapy-induced peripheral neuropathy. | Neuromodulation; 2026 | Reports the effect of SCS on QST (monitoring), not baseline prediction of response |

*Summary of exclusion reasons: conference abstract, not peer-reviewed (n = 9); registry protocol reporting no results (n = 1); effect / monitoring biomarker rather than prediction of response (n = 2).*
