# Supplementary material for: Neurophysiological and Psychophysical Biomarkers for Predicting Spinal Cord Stimulation Treatment Response in Chronic Neuropathic Pain: A Systematic Review
Source: J Pers Med. 2026 Sep 22;16(10):486. doi: 10.3390/jpm16100486 (PMC13706508; doi:10.3390/jpm16100486)
Supplement: Supplementary file 1 [file jpm-16-00486-s001.zip › Supplementary File S3. PRISMA 2020 Checklist.pdf]

Supplementary File S3. PRISMA 2020 Checklist

Neurophysiological and Psychophysical Biomarkers for Predicting Spinal Cord Stimulation Treatment Response in Chronic Neuropathic Pain: A Systematic Review · PROSPERO CRD420261410302

Section / Topic	Item #	Checklist item	Location where reported
**TITLE**			
Title	1	Identify the report as a systematic review.	Title page — "…: A Systematic Review".
**ABSTRACT**			
Abstract	2	See the PRISMA 2020 for Abstracts checklist.	Structured abstract (Background/Objectives/Methods/Results/Conclusions).
**INTRODUCTION**			
Rationale	3	Describe the rationale in the context of existing knowledge.	§1 Introduction, paras 1–3.
Objectives	4	Provide an explicit statement of the objective(s) or question(s).	§1 Introduction, para 4; Abstract (Objectives).
**METHODS**			
Eligibility criteria	5	Specify inclusion/exclusion criteria and how studies were grouped.	§2.2 Eligibility criteria.
Information sources	6	Specify all databases, registers, and other sources; date last searched.	§2.3; deviations re: Google Scholar/PsycINFO in §2.8.
Search strategy	7	Present the full search strategies for all sources.	§2.3; Supplementary File S1 (full strategies).
Selection process	8	Specify methods to decide inclusion, how many reviewers, independence.	§2.4 (dual, independent, blinded; κ reported; R.F./F.A.; arbiter C.A.O.).
Data collection process	9	Specify methods to collect data, number of reviewers, independence.	§2.5 (duplicate, R.F./F.A.); author contact not undertaken (§2.8).
Data items	10a	List and define all outcomes sought.	§2.5; §3.3; Table 2. Secondary outcomes not synthesizable (§2.8).
Data items	10b	List and define all other variables sought.	§2.5; Table 1.
Study risk of bias	11	Specify methods to assess risk of bias, tools, number of reviewers.	§2.6 (QUIPS/PROBAST/QUAPAS, duplicate).
Effect measures	12	Specify effect measures used in synthesis/presentation.	§2.7; associations as r/OR/HR/AUC (Tables 2–3).
Synthesis methods	13a	Describe processes to decide which studies were eligible for each synthesis.	§2.7; grouped by biomarker domain.
Synthesis methods	13b	Describe methods to prepare data for synthesis.	§2.5, §2.7.
Synthesis methods	13c	Describe methods to tabulate/visually display results.	§2.7 (SWiM); Tables 1–3; Figures 1–2.
Synthesis methods	13d	Describe methods to synthesize results and rationale.	§2.7, §2.8 (SWiM; meta-analysis not feasible — <3 commensurate studies/domain).
Synthesis methods	13e	Describe methods to explore heterogeneity.	§3.3, §4 (narrative).
Synthesis methods	13f	Describe sensitivity analyses.	Not performed — not feasible without meta-analysis (§2.8).
Reporting bias	14	Describe methods to assess risk of bias due to missing results.	Funnel plots/Egger's not performed (<10 studies/synthesis); considered narratively (§2.8, §4).
Certainty assessment	15	Describe methods to assess certainty in the body of evidence.	GRADE adapted, applied narratively; formal SoF tables not produced (§2.8).
**RESULTS**			
Study selection	16a	Report numbers screened/included with a flow diagram.	§3.1; Figure 1 (PRISMA flow).
Study selection	16b	Cite studies excluded at full text with reasons.	§3.1; Supplementary File S2.
Study characteristics	17	Cite each included study and present characteristics.	§3.2; Table 1.
Risk of bias in studies	18	Present risk-of-bias assessments for each study.	§3.5; Figure 2.
Results of individual studies	19	Present per-study summary statistics and effects.	Tables 2–3.
Results of syntheses	20a	Summarize characteristics/risk of bias of contributing studies.	§3.2–3.5.
Results of syntheses	20b	Present results of statistical/narrative syntheses.	§3.3–3.4 (by domain).
Results of syntheses	20c	Present results of heterogeneity investigations.	§3.3, §4.
Results of syntheses	20d	Present sensitivity analyses.	Not applicable (§2.8).
Reporting biases	21	Present assessments of risk of bias due to missing results.	§2.8, §4 (narrative; formal tests infeasible).
Certainty of evidence	22	Present certainty in the body of evidence per outcome.	§3.5, §4 — certainty low to very low (narrative; §2.8).

**DISCUSSION**			
Discussion	23a	Provide a general interpretation of the results.	§4 Discussion.
Discussion	23b	Discuss limitations of the evidence.	§4 (limitations).
Discussion	23c	Discuss limitations of the review processes.	§4; §2.8 (protocol deviations).
Discussion	23d	Discuss implications for practice, policy, and research.	§4, §5 Conclusions.
**OTHER INFORMATION**			
Registration and protocol	24a	Provide registration information including register name and number.	PROSPERO CRD420261410302 (title page, §2.1).
Registration and protocol	24b	Indicate where the protocol can be accessed.	PROSPERO record; §2.1.
Registration and protocol	24c	Describe amendments to information provided at registration.	§2.8 Deviations from the Registered Protocol.
Support	25	Describe sources of financial/non-financial support and role of funders.	Funding statement (back matter).
Competing interests	26	Declare any competing interests.	Conflicts of Interest (back matter).
Availability of data	27	Report which materials are publicly available and where.	Data Availability Statement; Supplementary Files S1–S3.

Reference: Page MJ, McKenzie JE, Bossuyt PM, et al. The PRISMA 2020 statement. BMJ 2021;372:n71. Deviations from the registered protocol are documented in Section 2.8 of the manuscript.
