# Supplementary material for: Neurophysiological and Psychophysical Biomarkers for Predicting Spinal Cord Stimulation Treatment Response in Chronic Neuropathic Pain: A Systematic Review
Source: J Pers Med. 2026 Sep 22;16(10):486. doi: 10.3390/jpm16100486 (PMC13706508; doi:10.3390/jpm16100486)
Supplement: Supplementary file 1 [file jpm-16-00486-s001.zip › Table S1. full funding and conflict-of-interest disclosures for each included study.pdf]

Supplementary Table S1. full funding and conflict-of-interest disclosures for each included study

Full disclosures as reported in each source publication; industry involvement was declared in several device-focused studies.

Study	Funding	Conflicts of interest
**Sindou 2003**	NR	NR
**Pluijms 2015**	Funded (source not stated)	NR
**Chen 2024**	National Natural Science Foundation of China (81901146, 82271259); Excellent Youth Foundation of Hunan; Key Laboratory of Hunan Province (2018TP1009); Huizhiyucai Project, Third Xiangya Hospital, Central South University	None declared
**Mironer 2000**	NR	NR
**Eisenberg 2006**	NR	NR
**Campbell 2015**	None	None declared
**Ramaswamy 2019**	NR	NR
**El-Sayed 2026**	Canadian Institutes of Health Research (CIHR) and the Mayday Fund	A. Bhatia has received consultancy fees from Bioventus, Boston Scientific and Medtronic; his institution received Medtronic funding for neuromodulation outcome studies
**Witjes 2025**	CIHR postdoctoral fellowship (2015-2016/358832); Quebec Pain Research Network; Stichting Neurobionics Foundation (unrestricted MEG grants)	F.J.P.M. Huygen: grants/personal fees from Abbott and Saluda Medical; personal fees from Pfizer and Grünenthal; independent support from ZonMw
**Gopal 2025**	US National Institutes of Health (NIH) award R00NS119672	J.G. Pilitsis is medical advisor for Aim Medical Robotics with stock equity; several co-authors are employees of NuVasive Clinical Services
**Dunn 2026**	US National Institutes of Health (NIH) award R00NS119672	Authors from the same group report industry ties (see Gopal 2025); specific disclosures per the source publication
**Levy 2024**	Saluda Medical (study sponsor)	Numerous authors report consulting fees, grants and/or stock from neuromodulation device manufacturers including the sponsor Saluda Medical; N. Soliday, R.V. Duarte and A. Leitner are Saluda Medical employees; remaining authors none

NR, not reported. Where the source stated only that conflicts were declared without listing them, this is indicated.
